# Human coronavirus OC43 infection in human cerebral organoids: novel insights on pathogenesis and potential therapeutic interventions

**DOI:** 10.1186/s12929-025-01193-z

**Published:** 2025-11-05

**Authors:** Juntong Liu, Yao Deng, Weibang Huo, Jingdong Song, Chengcheng Zhai, Lan Wei, Changcheng Wu, Gaoqian Zhang, Baoying Huang, Wenling Wang, Roujian Lu, Na Zhu, Wenjie Tan

**Affiliations:** National Key Laboratory of Intelligent Tracking and Forecasting for Infectious Diseases, National Institute for Viral Disease Control and Prevention, Chinese Center for Disease Control and Prevention, Beijing, 100052 China

**Keywords:** Human coronavirus, Human cerebral organoids, Inflammation, Pathogenesis, Therapy

## Abstract

**Background:**

Since the COVID-19 pandemic, there has been a documented rise in the incidence of neurological manifestations among individuals complicated with encephalitis or myelitis. The spectrum of neurological symptoms associated with HCoVs infections is expanding. However, the infection characteristics and pathogenesis of seasonal HCoVs to the central nervous system remain obscure. No pharmacological agents have demonstrated the capacity to specifically and efficaciously mitigate the neurological symptoms induced by HCoVs infections to date.

**Methods:**

We developed human cerebral organoids (HCOs) derived from human induced pluripotent stem cells and established a blood–brain barrier (BBB) HCOs co-culture model. We subjected these models to seasonal human coronavirus (HCoV) infections to investigate the viral characteristics within the central nervous system (CNS). Utilizing RNA sequencing, we conducted a preliminary exploration of the mechanisms underlying virus-induced inflammatory responses in the CNS. Furthermore, we assessed the efficacy of antiviral and anti-inflammatory drugs using the HCO model.

**Results:**

Our results showed that among seasonal coronaviruses, HCoV-OC43 replicates efficiently within the organoids, primarily targeting neurons and astrocytes, and disrupts the barrier function of the BBB. RNA sequencing analysis revealed that HCoV-OC43 infection triggers an inflammatory response through the TNF and NF-κB signaling pathways, leading to cell death, impaired neuronal function, and disrupted interneuron signaling. Interestingly, Bardoxolone methyl (CDDO-Me) demonstrated antiviral effects comparable to remdesivir, reducing both inflammation and cell death.

**Conclusions:**

Conclusively, HCOs infected with HCoV-OC43 offer valuable insights into the pathogenesis of HCoVs in central nervous system (CNS), and might serve as a tool for developing novel therapeutic strategies for HCoVs infections, including COVID-19, especially on exploring treatment candidates.

**Graphical abstract:**

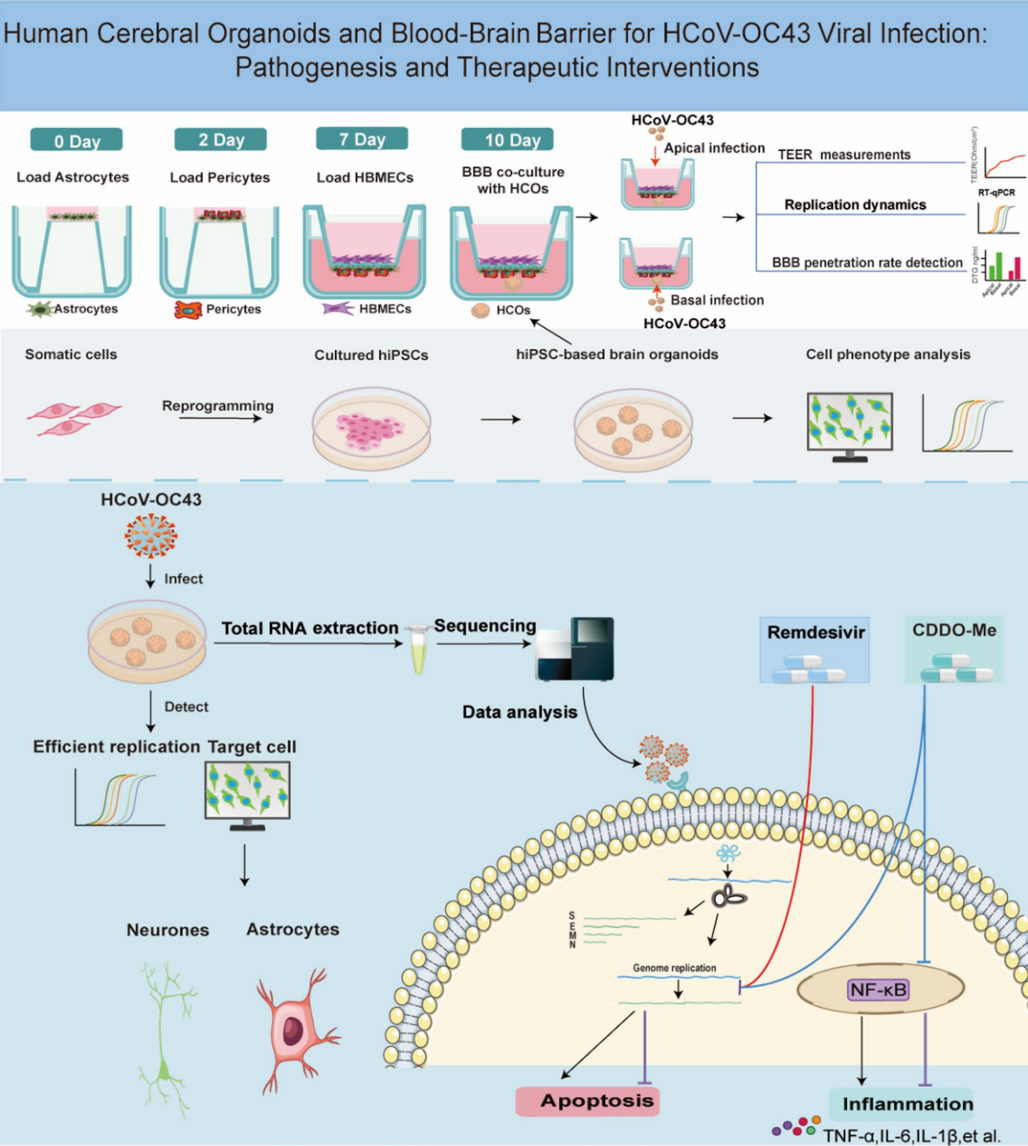

**Supplementary Information:**

The online version contains supplementary material available at 10.1186/s12929-025-01193-z.

## Introduction

Human coronaviruses (HCoVs) primarily cause respiratory infections; however, studies have shown that several HCoVs cause neurological symptoms and the burden of disease is often grossly overlooked[1]. HCoVs RNA has been detected in the brain tissue of patients with viral encephalitis or multiple sclerosis (MS) [2, 3]. Autopsy studies of 44 adults with COVID-19 showed 40 cases were positive with viral RNA and both in-situ hybridization and immunofluorescence confirmed the presence of viral antigen in neurons [4]. It is worth noting that the common and most representative symptoms of COVID-19 (including Long COVID-19) caused by SARS-CoV-2 include fatigue or muscle weakness, discomfort, difficulty breathing, headache, dizziness or "brain fog," depression, irritability, frustration, insomnia, and many other neurological disorders [5]. In animal models, A high rate of SARS-CoV-2 neuronal invasion was found in transgenic mice expressing human angiotensin-converting enzyme 2 (ACE2), which is associated with neuronal cell death and encephalitis [6]. In another study, microscopic imaging of brain samples from non-human primates infected with SARS-CoV-2 revealed direct neurological damage, identifying neurons as the primary targets. This damage can subsequently lead to neuroinflammation and the destruction of blood vessels following infection [7]. A seasonal coronavirus, HCoV-OC43, which belongs to the same β-coronavirus genus as SARS-CoV-2, has been linked to cases of encephalitis following infection [8–12]. Clinical data indicate HCoV-OC43 infection can result in acute neurological dysfunction in children, and exposure to HCoV-OC43 has been linked to long-term neurological disorders in adults [13–17]. Both HCoV-OC43 and another seasonal human coronavirus 229E(HCoV-229E) are reported to induced persistent infection in human neuronal cells, oligodendrocytes, and glial cell lines, characterized by severe cytopathic effects on these cells [18–20]. Infection of mice with HCoV-OC43 induces neuroinflammation, resulting in severe neuropathological changes primarily characterised by flaccid paralysis and demyelination [21–23]. These findings suggest that HCoVs exhibit neurotropism, and the pathogenesis of CNS diseases caused by HCoVs infection and novel therapeutic strategies should be explored. However, no pharmacological agents have demonstrated the capacity to specifically and efficaciously mitigate the neurological symptoms induced by HCoVs infections to date.

Current research on the susceptibility and pathogenicity of HCoVs infections in the central nervous system (CNS) is primarily limited to two-dimensional (2D) cell cultures and animal models. However, both of these approaches have certain limitations. The 2D cell cultures lack the organizational structure, cellular interactions, and cellular complexity [24, 25]. Additionally, neural structures and functions considerably differ between experimental animals and humans [26]. Therefore, there is an urgent need for a more physiological model to study the neurovirulence of coronaviruses.

Human induced pluripotent stem cell (hiPSC)-derived human cerebral organoids (HCOs) grow in a three-dimensional (3D) manner in cell cultures. Compared with traditional 2D cell cultures and animal models, HCOs effectively mimic human brain development and function due to their diverse cell types, structures, and developmental stages [27]. In virology research, HCOs overcome the limitations of virus-specific host infections, making them suitable for studying viral tropism and the receptors essential for infection [28]. Additionally, HCOs offer a more accurate reflection of the interactions between viruses and human tissues and cells. Currently, HCOs have been used to investigate the infection and pathogenic mechanisms of viruses such as Zika virus (ZIKV), Herpes simplex virus type I (HSV-1), Severe acute respiratory syndrome coronavirus 2 (SARS-CoV-2), and the Japanese encephalitis virus (JEVs) [29–32]. However, a comprehensive understanding of the pathogenesis of HCoVs-driven (especially seasonal coronaviruses-driven) manifestations in CNS based on HCOs is still largely elusive.

In this study, we developed HCOs derived from hiPSC and subsequently infected them with seasonal HCoVs. Our results showed that, among all human seasonal HCoVs tested, HCoV-OC43 primarily replicates effectively in HCOs. This is the first time HCoV-OC43, a seasonal coronavirus, has been identified to infect and replicate directly in a human physiological model that mimics the human central nervous system, and it has demonstrated direct neurovirulence.

HCOs offer valuable insights into the neuroinflammatory pathology of HCoV-OC43 and might serve as a tool for developing novel therapeutic strategies for CNS complications caused by HCoVs infections, including COVID-19.

## Material and methods

### hiPS cell lines culture

Human induced pluripotent stem cells (hiPSCs) were purchased from Cauliscell Biotechnology, seeded onto 6-well plates coated with 10 μg/µL Vitronectin XFTM (STEMCELL Technologies, 07180) and maintained in mTesR Plus medium (STEMCELL Technologies, 100–0276). Cells were fed daily and passaged every 4–5 days using the Gentle Cell Dissociation Reagent (STEMCELL Technologies, 100–0485) solution treatment, and mechanical dissociation.

### Generation of HCOs

As described by the Lancaster MA team and STEMCELL Technologies, we differentiated HCOs [27]. Initially, hiPSCs were dissociated into single-cell suspensions using the Gentle Cell Dissociation Reagent. Then, we seeded 100 µL of STEMdiff Cerebral Organoid Basal Medium supplemented with Supplement A into a 96-well plate (9000 cells/well) and 20 μM Rho-associated protein kinase (ROCK) inhibitor.

On the second and fourth consecutive days of cultivation, we added 100 µL of STEMdiff Cerebral Organoid Basal Medium supplemented with Supplement A (STEMCELL Technologies, 08570). On day 5, we replaced the medium with STEMdiff Cerebral Organoid Basal Medium supplemented with Supplement B. On day 7, we embedded the embryoid bodies in Matrigel (Corning, 354,277) and added STEMdiff Cerebral Organoid Basal Medium supplemented with C and D to sustain cultivation for three days. On day 10, we replaced the medium with mature cerebral organoids, STEMdiff Cerebral Organoid Basal Medium supplemented with E, and placed it on a fixed-track rocker for sustained cultivation.

### Viral infections

The HCoV-OC43 strain (VR1558, ATCC), HCoV-NL63 strain (Amsterdam, NC_005831), HCoV-229E strain (VR740, ATCC), HCoV-HKU1 strain (NO. KT779555, this strain was isolated from our laboratory) and ZIKV (SMGC-1) were used in this study. HCOs (Day 45) were transferred to 24-well plates with one organoid per well and co-incubated with 100 TCID_50_ viral liquid for 6 h. After one PBS wash, 500 µL of mature culture medium was added to sustain cultivation. For the antiviral and anti-inflammatory treatment groups, the corresponding drug concentration medium was substituted accordingly (Remdesivir 100, 10, 1, 0.1, and 0.01 μM; NEC-1, 40 µM; CDDO-Me, 0.8 µM).

### Median tissue culture infective dose (TCID_50_)

A 10 µl of the virus to be tested was subjected to tenfold serial dilution, ranging from 10^–1^ to 10^–8^. Following dilution, the samples were sequentially inoculated into a 96-well plate filled with a monolayer of MRC5 cells, with eight replicate wells for each dilution, and 100 µL of virus diluent was added to each well. A control group was established and incubated in a culture chamber at 37 °C with 5% CO_2_ for 2 h. After adsorption, the supernatant was discarded, the wells were washed once with PBS, and 100 µL of virus maintenance solution was added to sustain cultivation. Daily observations of cytopathic effects (CPE) were performed under a microscope for 4–5 days, and the CPE status of each well was recorded. The TCID_50_ was calculated using the Reed-Muench method.

### Real-time quantitative reverse transcription polymerase chain reaction (RT-qPCR)

RNA extraction and RT-qPCR analysis were conducted for each condition and time point. Nucleic acids within the cells of the HCOs were extracted according to the manufacturer's protocol using the Heaven's Root Cell Nucleic Acid Extraction Kit. cDNA synthesis was performed with SuperScript III and Random Hexamers using 500 ng–1 μg of total RNA according to the manufacturer’s instructions. The TB Green Premix Ex TaqTM II (Takara, RR820A) kit was employed to detect the viral nucleic acid, TJU1, MAP2, PAX6, CTIP2, TBR1, TBR2, TNF-α, MCP-1, IL-1β, IL-6, IL-8, IL-12, caspase 3, Fas, and Apaf1 relative expression levels, following the manufacturer's instructions. Quantification was performed in Excel, with actin as the reference gene for calculating ΔCt values and untreated samples as calibrators for computing ΔΔCt values. Data were presented as fold change (2^−ΔΔCt^). Primers used for RT-qPCR are listed in Table S1.

Nucleic acids were extracted using the Cell Culture Supernatant Nucleic Acid Extraction Kit. Subsequently, viral nucleic acid copy numbers were determined using the One Step PrimeScript RT-PCR kit (Takara, RR064A) and a LightCycler96 real-time fluorescence quantitative PCR instrument (Roche, Basel, Switzerland).

### Cell viability detection

In this study, the LDH method was used for cell viability detection. The LDH-Cytotoxicity Assay Kit was purchased from Abcam (ab65393), and the tests were conducted in accordance with the manufacturer's instructions.

### Transmission electron microscope

A solution containing 2% paraformaldehyde and 2.5% glutaraldehyde was used, followed by fixation with 1% osmium tetroxide, dehydration using graded ethanol, and embedding in PON812 resin. Sections (80 nm thick) were cut from the resin block and stained with uranyl acetate and lead citrate. Ultrathin sections were observed using transmission electron microscopy, and images were captured.

### Immunostaining of HCOs

For immunofluorescence staining, we used a 10% formalin solution to fix the HCOs infected with the virus for 4 days, allowing them to fixate for 24 h. The sections were embedded in paraffin to form blocks. Slices of 5 μm were prepared, baked at 60 °C for 1 h, and dewaxed in xylene I, II, III, and IV for 5 min each, followed by hydration in anhydrous ethanol I and II for 2 min each, 70% ethanol for 2 min, and immersion in ddH_2_O for 5 min. The slices were placed in antigen–antibody retrieval solution with a pH of 6.0 or 9.0 at a temperature exceeding 90 °C for 10 min, followed by natural cooling. After one wash with DPBS, the slices were soaked in 0.2% Triton for 10 min, blocked with 5% BSA for 1 h, and incubated overnight at 4 °C with the primary antibody prepared in 1% BSA. The following primary antibodies were used: anti-TUJ1(STEMCELL Technologies 60,052, 1:1,000), anti-MAP2 (BD Biosciences 556320,1:500), anti-CTIP2 (Abcam 18465,1:100), anti-SOX2 (Abcam 171380,1:200), anti-Ki-67 (NOVUS-NBP1-31,231,1:1,000), anti-GFAP (Abcam 4648,1:100), and anti-HCoV-OC43 NP (SinoBiological 40643-T62,1:500). Subsequently, the slices were rinsed 5 times with DPBS for 5 min each, and incubated at room temperature for 1 h in a solution containing 1% BSA with secondary antibody preparation and DAPI. The following secondary antibodies were used: donkey anti-rat Alexa 488 (Invitrogen-A-21208, 1:1,000), donkey anti-rabbit Alexa 555(Invitrogen-A-31572, 1:1,000), donkey anti-mouse Alexa 647(Invitrogen-A-31571, 1:1,000), DAPI (Invitrogen-A-22287, 1:1,000). The slices were cleansed five times with DPBS, each immersion lasting 5 min, and 50 µL of anti-fade mounting medium was added for sealing. Images were captured using a confocal microscope (Leica TCS SP8, Germany).

### Quantification of apoptosis

Paraffin sections of the HCOs were used to detect apoptosis. The pretreatment procedure was consistent with the immunofluorescence protocol. Apoptosis was detected using the TUNEL BrightRed Apoptosis Detection Kit (Vazyme, A113-01) following the manufacturer's instructions. Images were captured using a confocal microscope (Leica TCS SP8, Germany).

### RNA sequencing

RNA sequencing and analysis for the HCoV-OC43 experiment, three replicates were used per condition and time point (1, 4, and 14 dpi). The TRIZOL method was employed to extract intracellular nucleic acids, with RNA samples undergoing stringent quality control using an Agilent 2100 bioanalyser to precisely assess RNA integrity. Given that most of the mRNA in eukaryotes possess a poly (A) tail, mRNA enriched with poly (A) tails was obtained via oligo (dT) magnetic bead enrichment. Subsequently, the mRNA was randomly fragmented using divalent cations in the NEB Fragmentation Buffer, followed by library construction using either NEB standard or strand-specific library preparation methods [33]. Upon completion of library construction, initial quantification was performed using the Qubit 2.0 Fluorometer, followed by library dilution to 1.5 ng/µL. Subsequent analysis of the library's insert size was conducted using the Agilent 2100 bioanalyser. Upon confirmation of the expected insert size, RT-qPCR was employed to accurately quantify the effective concentration of the library (with an effective concentration > 1.5 nM) to ensure library quality.

Once the libraries passed the quality control, they were pooled according to their effective concentrations and the desired amount of target data for Illumina sequencing.

### RNA-seq data analysis

Initially, the sequencing data underwent quality control. Upon qualification, HISAT2 software was utilised for the rapid and precise alignment of Clean Reads to the human reference genome, acquiring the positional information of reads on the reference genome. Based on the positional information of the gene alignment in the reference genome, the number of reads covering each gene (including newly predicted genes) from start to end was calculated. Reads with alignment quality values < 10, unpaired alignments, and reads aligned to multiple regions of the genome were filtered. This analysis utilised the feature count tool in the Subread software [34]. The union of differentially expressed genes from all comparison groups was considered the set of differentially expressed genes. Hierarchical clustering was applied to the FPKM values of the genes for clustering analysis, with normalisation performed on the rows (row) (Z-score). GO functional and KEGG pathway enrichments used a threshold of *padj* < 0.05 for significance enrichment [35]. Gene set enrichment analysis (GSEA) did not require a specified threshold for differential gene expression. Instead, genes were sorted based on their differential expression between the two sample groups, and statistical methods were employed to determine whether the predefined gene sets were enriched at the top or bottom of the sorted table.

### Establishment of a co-culture model of the blood–brain barrier (BBB) and HCOs

Human brain endothelium (HBEC) was purchased from ATCC (VA, USA; CRL-3245), human astrocytoma was purchased from ATCC (VA, USA; CRL-1718), and human brain vascular pericytes were purchased from iXCells Biotechnologies (San Diego, CA, USA; 10HU-031). We referred to the protocol for constructing the BBB by the teams of Sean N. Avedissian and Saoirse E. O 'Sullivan. Different types of cells were inoculated at the appropriate time. When the cells were cultured in Transwell (Corning, 3462) for 10 days, the HCOs were co-cultured with Transwell to obtain the co-cultured model [36, 37].

### Trans-epithelial electrical resistance (TEER) measurements

The resistance measuring probe was washed in 75% (v/v) ethanol, air-dried, and equilibrated in endothelial medium for 15 min inside a biosafety cabinet. After equilibration, the probe was positioned in the Transwell insert well, with the shorter arm placed just above the HBECs on the apical layer and the longer arm just above the coverslip at the bottom of the well. To ensure consistent measurements, the probe position was kept identical across all Transwell plates, and at least three positions per Transwell were measured to determine TEER. TEER values were recorded daily until completion of the experiment.

### Detect the penetration rate of FITC-Dextran

A reliable method for measuring BBB permeability was conducted referenced Reka Natarajan’s group [38], DMEM/F12 (without phenol red), containing 100 µg/mL of FITC-Dextran, was applied to the apical side and incubated for 30 min. Subsequently, 10 µL of medium was collected from both the apical and basal compartments and transferred to a black 96-well plate, which had been pre-filled with 100 µL of PBS. The fluorescence intensity was measured at an excitation wavelength of 494 nm and an emission wavelength of 521 nm using a microplate reader. The permeability of FITC-Dextran was calculated from the fluorescence intensities using the following formula: permeability = (apical fluorescence intensity/basal fluorescence intensity) × 100%.

### Immunostaining of BBB

HCoV-OC43 infected BBB cells were fixed with 4% paraformaldehyde in situ for at least 8 h, permeabilized with 0.2% Triton X-100, blocked with 5% BSA, reacted with anti-GFAP protein antibody (CST 80788T, 1:100), claudin 5 antibody (ThermoFisher 35-2500,1:100), CD146 antibody (Abcam ab75769, 1:100). After incubation of the second antibody, the tablet was sealed and photographed. Samples were detected and recorded under a laser scanning confocal microscope system (Leica TCS SP8, Germany).

### Statistical analysis

Data are expressed as mean ± SD with distinct dots for each measurement. An unpaired t-test was used to compare the differences between two groups. One-way ANOVA was used to compare three or more groups. *p* < 0.05 indicates significant difference. Statistical analyses were performed using GraphPad Prism 9.5.

## Results

### Development and validation of HCOs

To better understand the neurotropism of HCoV-OC43, we generated HCOs using hiPSCs, as previously established by the Lancaster MA team [27] (Supplemental Fig. 1a). These organoids had dorsal forebrain region specifications and contained ventricle-like structures formed by SOX2^+^ neural progenitors (apical radial glia) and Ki-67^+^ proliferating cell. Early corticoid structures marked by MAP2^+^ mature neurons, TUJ1^+^ immature neurons and CTIP2^+^ deep cortical lamina neurons were observed. These organoids also contained GFAP^+^ astrocytes (Supplemental Fig. 1b). We observed an increase in the relative mRNA expression levels of various subtypes of neuronal markers, such as *MAP2*, *TUJ1*, *CTIP2*, *TBR1*, *TBR2*, and the apical radial glial cell marker *PAX6* mRNA (Supplemental Fig. 1c). These results show that our HCOs contain major cell types that mark early human brain development and can, therefore, serve as an in vitro model for CNS infection with viruses.

### HCoV-OC43 can replicate effectively within HCOs, targeting both neurons and astrocytes

**Fig. 1:**
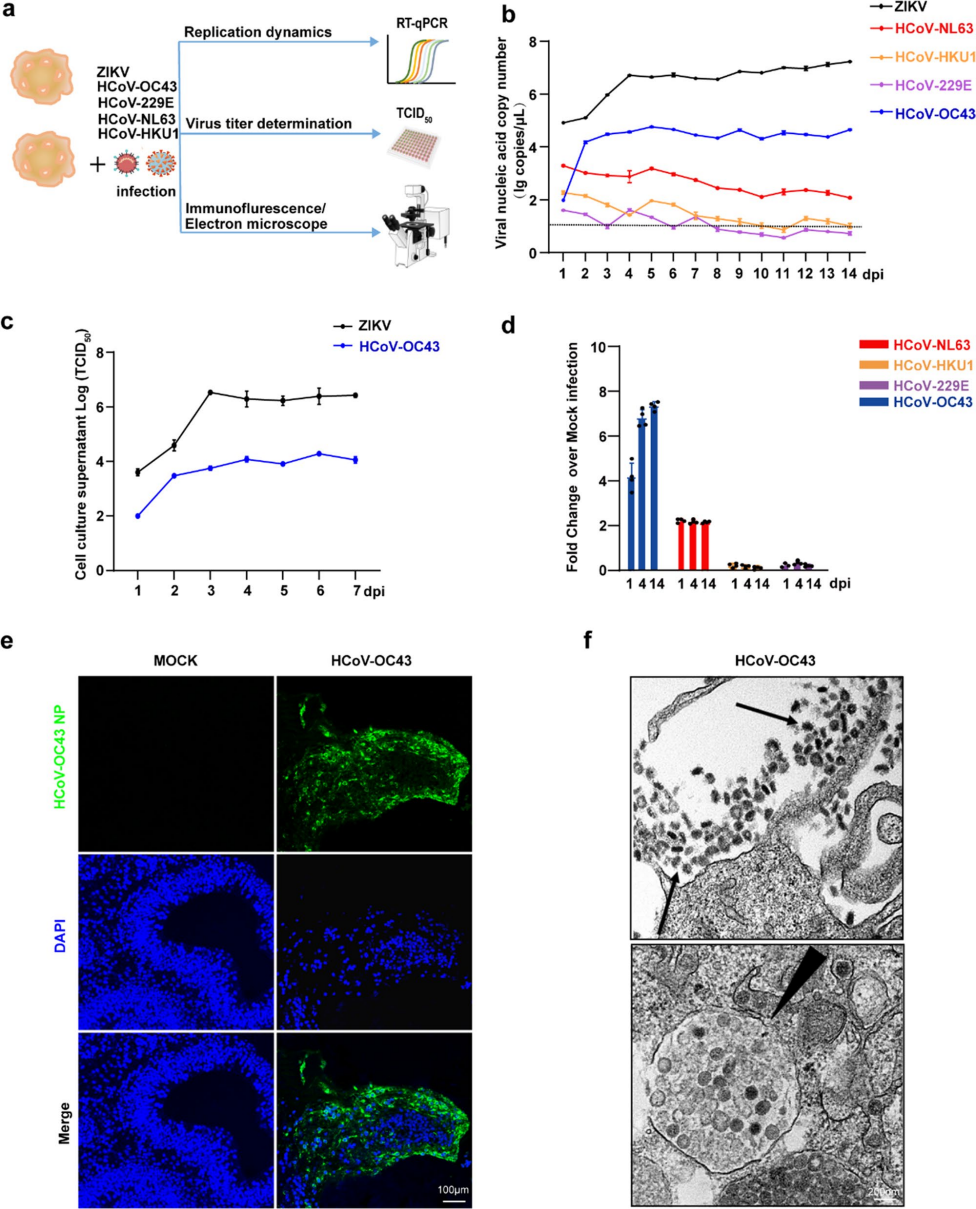
HCoV-OC43 can infect and replicate efficiently in HCOs. **a**. Schematic overview of the experimental set-up. **b**. Viral nucleic acid copy numbers detected in cell culture supernatant following HCoV-OC43, HCoV-NL63, HCoV-229E, and HCoV-HKU1 infection of HCOs. **c**. Virus TCID_50_ was detected in cell culture supernatant after HCoV-OC43 infection of HCOs. **d**. Relative changes in intracellular viral nucleic acid copy numbers at 1-, 4-, and 14-days post-infection (dpi) in HCoV-OC43, HCoV-NL63, HCoV-229E, and HCoV-HKU1 infected HCOs. **e**. Expression of the NP protein (Green) in OC43-infected HCOs after 4 days, detected using immunofluorescence. **f**. Virus particles observed in OC43-infected HCOs after 4 days using transmission electron microscopy

To balance cellular complexity and reduce necrosis during long-term culture, 45-day-old HCOs were used for all infection experiments. Four seasonal coronaviruses (HCoV-OC43, HCoV-NL63, HCoV-229E, and HCoV-HKU1) and ZIKV were inoculated into HCOs, and multiple detection methods were employed to assess infection characteristics **(**Fig. 1a**)**. The successful replication of ZIKV in HCOs confirms that the HCOs generated by our group are suitable for virological research **(**Fig. 1b, c**)**. In culture supernatants, HCoV-OC43 RNA gradually increased, peaking at day 5 before stabilizing, whereas no significant differences were detected for the other three coronaviruses within 14 days (Fig. 1b). Viral titration assays further demonstrated that HCoV-OC43 titres rose with prolonged infection (Fig. 1c). For HCoV-OC43, viral RNA levels in HCO cells progressively increased at 1-, 4-, and 14-days post-infection (dpi). Conversely, no significant changes were observed following HCoV-NL63, HCoV-229E, or HCoV-HKU1 infection (Fig. 1d). Accordingly, HCoV-OC43 NP protein was detected in infected HCOs but not in mock-treated controls (Fig. 1e). Moreover, ultrastructural analysis of thin sections revealed extracellular virions and inclusion bodies filled with viral particles enclosed in cytoplasmic membrane-bound vesicles (Fig. 1f). The observed morphology is characteristic of the Coronaviridae family. Collectively, these results demonstrate that HCoV-OC43 can efficiently infect and replicate within HCOs.

**Fig. 2:**
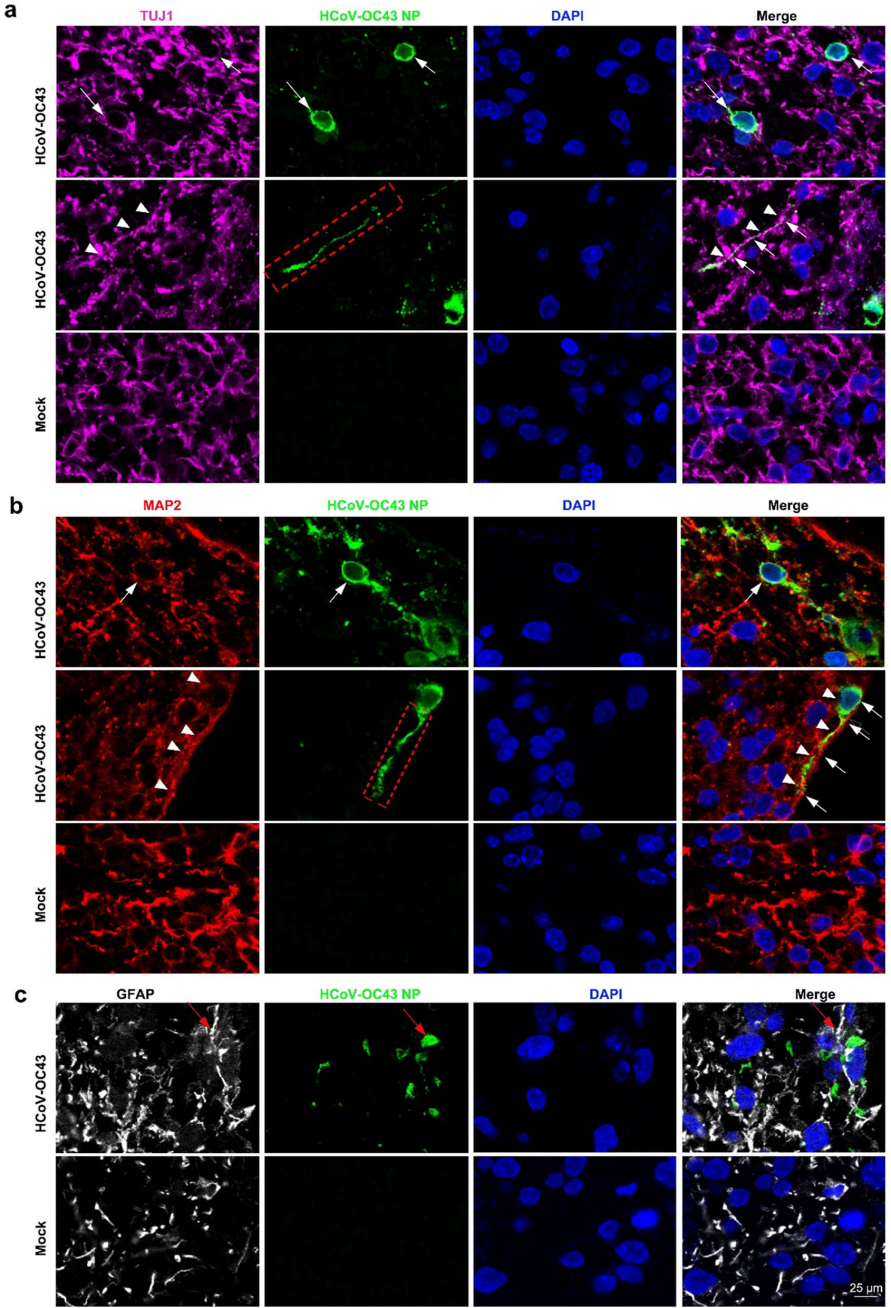
HCoV-OC43 infects HCOs target cells. **a** The immunofluorescence results were observed immature neuronal marker TUJ1 (Pink) show NP(Green) staining. **b** The immunofluorescence results were observed mature neuronal marker MAP2 (Red) show NP(Green) staining. **c** The immunofluorescence results were observed astrocyte marker GFAP (White) show NP(Green) staining

Next, we investigated the cell types targeted by HCoV-OC43 and their susceptibility to infection. TUJ1, a marker of immature neurons, TUJ1-positive cells show NP staining, indicating that HCoV-OC43 targeted immature neurons in these HCOs (Fig. 2a). Similarly, MAP2, a marker of mature neurons, MAP2-positive cells show NP staining, suggesting that HCoV-OC43 infects mature neurons (Fig. 2b). As indicated by arrows and triangles in the figure, we also observed the distribution of viral nucleocapsid proteins along the axons of neurons, and the discontinuous dotted distribution of tubulin in infected neurons (Fig. 2a, b). Glial fibrillary acidic protein (GFAP), a marker for astrocytes, GFAP-positive cells show NP staining, indicating astrocyte infection by HCoV-OC43 (Fig. 2c). However, no colocalization of the HCoV-OC43 NP protein with the apical radial glial cell marker SOX2 was observed (Supplemental Fig. 2). Similar to SARS-CoV-2, both target infected neurons and astrocytes [39, 40], but syncytium appeared after SARS-CoV-2 infected nerve cells [41], no syncytium was found after HCoV-OC43 infection.

### HCoV-OC43 infection in HCOs induces transcriptional dysregulation, potentially activating the TNF and NF-κB signaling pathways

**Fig. 3:**
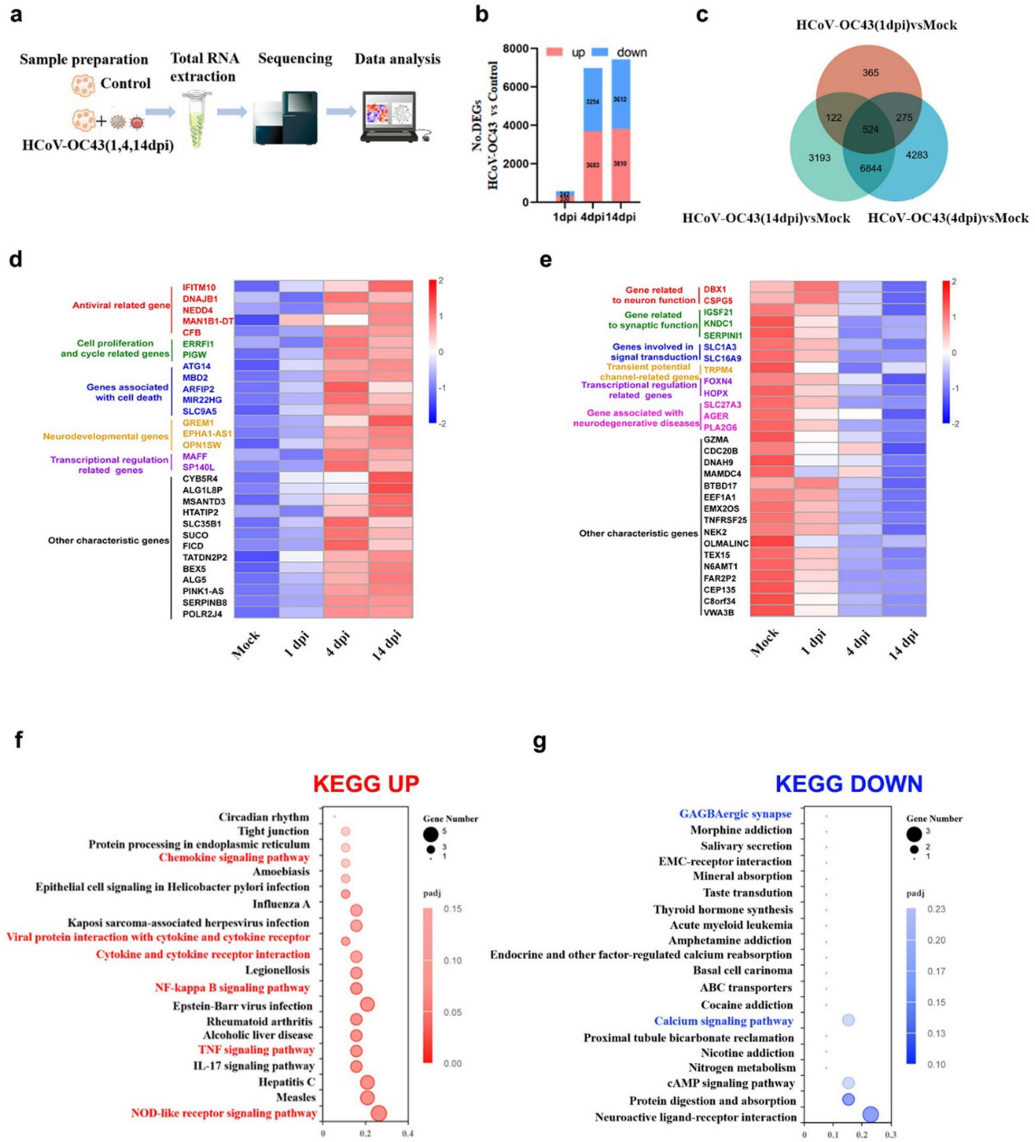
HCoV-OC43 infection of HCOs results in dysregulation of intracellular RNA-Seq. **a**. Overview of the RNA-seq flow chart of HCoV-OC43-infected HCOs. **b**. Graphs showing the number of differentially expressed genes (DEGs) in virus-infected HCOs. **c**. Venn diagram showing the DEGs in HCoV-OC43-infected HCOs at 1, 4, and 14 dpi. **d**. Top 30 upregulated DEGs (*padj* < 0.05) in HCoV-OC43-infected HCOs. **e**. Top 30 downregulated DEGs (*padj* < 0.05) in HCoV-OC43-infected HCOs. **f**. Top 20 KEGG pathways associated with upregulated genes (*padj* < 0.05) in HCoV-OC43-infected HCOs. **g**. Top 20 KEGG pathways associated with downregulated genes (*padj* < 0.05) in HCoV-OC43-infected HCOs (n = 3)

To investigate the molecular characteristics of HCoV-OC43 infection in HCOs, we performed RNA-seq analysis of the transcriptomes of the infected cultures (Fig. 3a). At 1 dpi, we identified 572 differentially expressed genes (DEGs), with 330 upregulated and 242 downregulated genes. At 4 dpi, there were 6,977 DEGs, including 3,683 upregulated and 3,294 downregulated genes. At 14 dpi, 7,422 DEGs were identified, of which 3,810 were upregulated and 3,612 were downregulated (Fig. 3b). Among these, 524 DEGs were common across all three time points (Fig. 3c). In the notably upregulated DEGs, we observed an enrichment of genes involved in antiviral responses, such as *IFITM10*, *DNAJB1*, *NEDD4*, *MAN1B1-DT*, and *CFB*; genes associated with cell proliferation and the cell cycle, such as *ERRFI1* and *PIGW*; genes related to cell death, such as *ATG14*, *MBD2*, *ARFIP2*, *MIR22HG*, and *SLC9A5*; genes implicated in neural development, such as *GREM1*, *EPHA1-A*, and *OPN1SW*; genes involved in transcriptional regulation, such as *MAFF* and *SP140L* (Fig. 3d). Conversely, among the significantly downregulated DEGs, we found enrichment of genes associated with neuronal functions, such as *DBX1* and *CSPG5*; genes related to synaptic functions, such as *ICSF21*, *KNDC1*, and *SERPINI1*; genes involved in cell signaling, such as *SLC1A3* and *SLC16A9*; genes implicated in transcriptional regulation, such as *FOXN4* and *HOPX*; genes associated with neurodegenerative diseases, such as *SLC27A3*, *AGER*, and *PLA2G6* (Fig. 3e). Furthermore, KEGG pathway enrichment analysis revealed significant activation of pathways related to inflammation, such as TNF, NF-κB, NOD-like receptor, chemokine signaling, and viral protein interactions with cytokines and cytokine receptors (Fig. 3f). In contrast, GABAergic synaptic and calcium signaling pathways were downregulated (Fig. 3g). Additionally, enrichment analysis of DEGs associated with inflammation and cell death showed significant upregulation of these genes as the infection progressed (Supplemental Fig. 3). These RNA-seq findings suggest that HCoV-OC43 infection in HCOs activates inflammatory responses through the NOD, TNF and NF-κB signaling pathway, leading to cell death, impaired neuronal and synaptic functions, and disrupted interneuronal signaling.

### HCoV-OC43 infection activates an inflammatory response and induces cellular apoptosis in HCOs

**Fig. 4:**
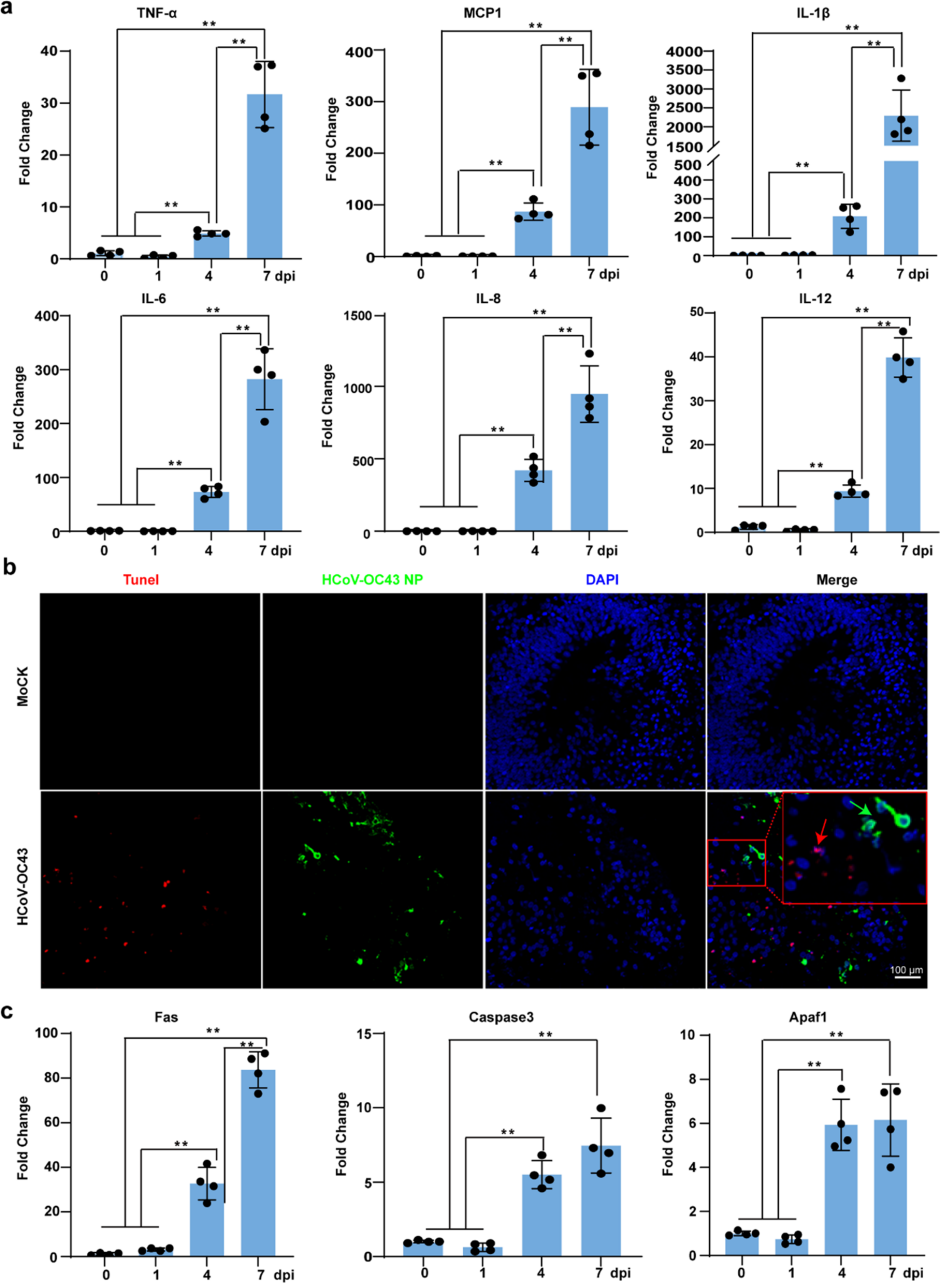
HCoV-OC43 infection of HCOs induces inflammation and cell death. **a**. RT-qPCR was used to detect the mRNA expression levels of intracellular inflammatory factors (TNF-α, MCP1, IL-1β, IL-6, IL-8, and IL-12) after HCoV-OC43 infection of HCOs at 1, 4, and 7 days (*p* < 0.05). **b**. TUNEL method was used to detect cell death in HCOs after 4 days of HCoV-OC43 infection. **c**. RT-qPCR was used to detect the mRNA expression levels of apoptosis-related genes *caspase 3*, *Fas*, and *Apaf1* after HCoV-OC43 infection of HCOs at 1, 4, and 7 days (*p* < 0.05) (n = 4)

To validate the inflammatory response and cellular damage triggered by HCoV-OC43, we observed a significant increase in the relative expression levels of proinflammatory cytokines, including *TNF-α*, *MCP-1*, *IL-1*β, *IL-6*, *IL-8*, and *IL-12* mRNA, following HCoV-OC43 infection in HCOs (Fig. 4a). Additionally, we detected a substantial increase in cell death post-infection (Fig. 4b). Interestingly, non-HCoV-OC43 NP^+^ cells were TUNEL^+^, suggesting that HCoV-OC43 exerts an indirect, non-cell-autonomous bystander effect, leading to the death of uninfected cells. Furthermore, we detected a significant increase in the expression of apoptosis-related genes, including *caspase3*, *Fas*, and *Apaf1* (Fig. 4c). Collectively, these results indicate that HCoV-OC43 infection of HCOs activates inflammatory responses and induces cell death.

### Remdesivir and CDDO-Me effectively inhibit HCoV-OC43 infection as well as inflammation response and cellular apoptosis in HCOs

**Fig. 5:**
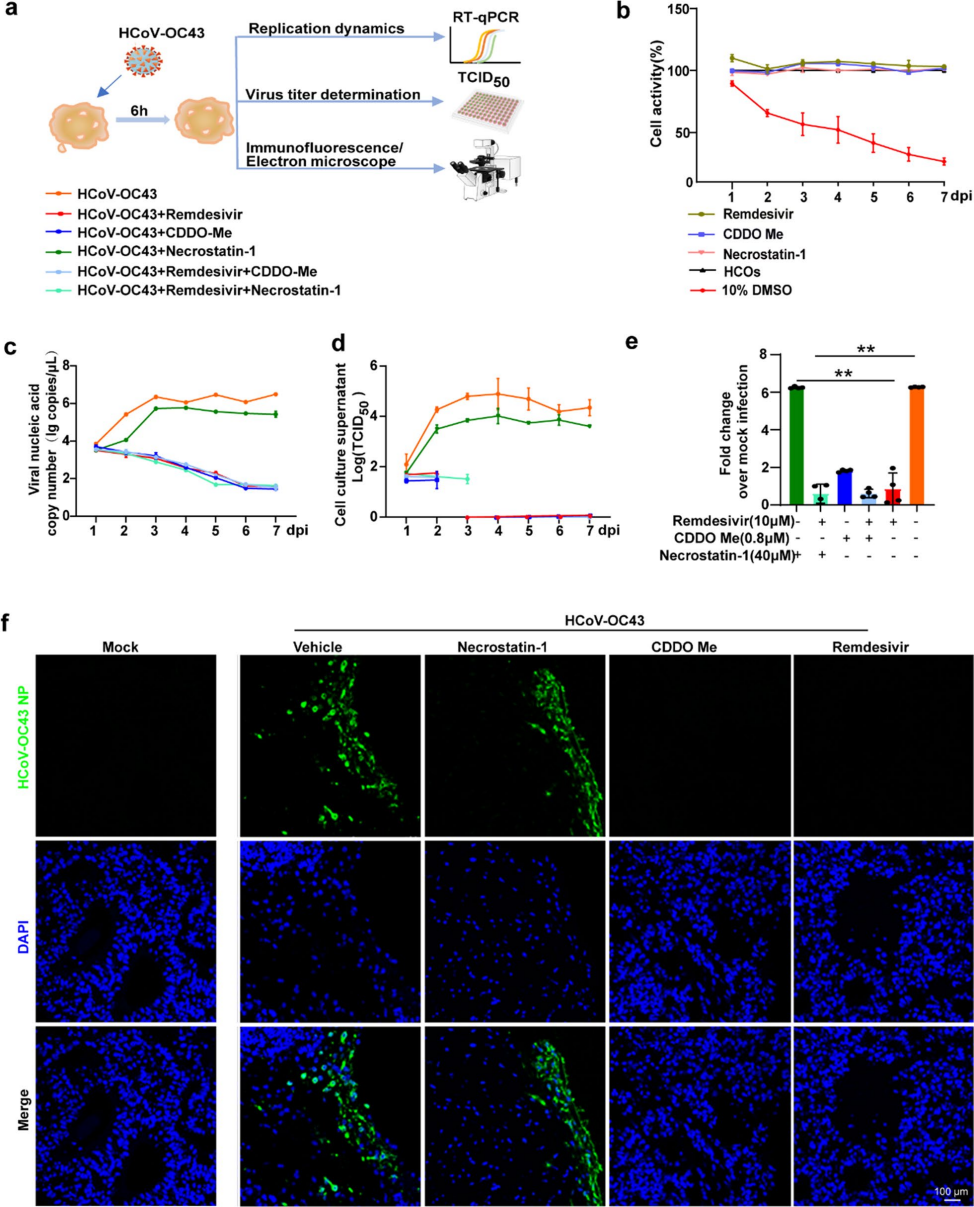
Remdesivir and CDDO-Me effectively inhibit HCoV-OC43 infection in HCOs. **a**. Schematic overview of the drug antiviral experimental set-up. **b**. The effects of remdesivir (100 µM), CDDO-Me (0.8 µM), necrostatin-1 (NCE1, 40 µM), 10% DMSO (positive control), and HCOs (negative control) on HCOs cell viability detected using LDH assay. **c**. Viral nucleic acid copy number in the cell culture supernatant after HCoV-OC43 infection of HCOs with the drug alone or in combination. **d**. Virus TCID_50_ detected in the cell culture supernatant after HCoV-OC43 infection of HCOs with the drug alone or in combination. **e**. The relative changes in intracellular viral nucleic acid copy number after HCoV-OC43 infection of HCOs with the drug alone or in combination. **f**. The NP protein expression of HCoV-OC43-infected HCOs detected using immunofluorescence after treatment with remdesivir (1 µM), CDDO-Me (0.8 µM), and Necrostatin-1(NCE1, 40 µM) (n = 4)

To determine whether antiviral drugs can effectively inhibit HCoV-OC43 replication in HCOs and whether anti-inflammatory drugs can prevent the inflammatory response and cellular damage post-infection, we tested antiviral drugs along with two compounds targeting the TNF pathway, either alone or in combination: (1) necrostatin-1 (NEC-1), an RIP1-targeted inhibitor of TNF-induced necroptosis. (2) CDDO-Me, an NRF2 agonist that attenuates the NF-κB mediated inflammatory response (Fig. 5a). Initially, we evaluated the effect of maximum drug concentration on cell viability and showed that remdesivir (10 μM), NEC-1 (40 μM), and CDDO-Me (0.8 μM) exerted no effect on cellular viability (Fig. 5b). Subsequently, we measured the nucleic acid levels and viral titres in the cell culture supernatant following drug treatment and found that remdesivir, either alone and in combination, effectively inhibited HCoV-OC43 replication in the HCOs. The anti-inflammatory drug CDDO-Me alone significantly suppressed HCoV-OC43 replication, whereas NEC-1 did not affect viral replication (Fig. 5c,d). Simultaneously, we assessed the relative expression levels of viral nucleic acids in HCOs cells. Treatment with remdesivir and CDDO-Me, either alone or in combination, reduced viral nucleic acid levels, whereas NEC-1 alone did not affect viral nucleic acid copy numbers compared with the virus control group (Fig. 5e). The antiviral effect of remdesivir was dose-dependent (Supplemental Fig. 4). Immunofluorescence confirmed the strong antiviral effects of remdesivir and CDDO-Me (Fig. 5f). Collectively, these findings indicate that remdesivir and CDDO-Me effectively inhibit HCoV-OC43 infection in HCOs.

**Fig. 6:**
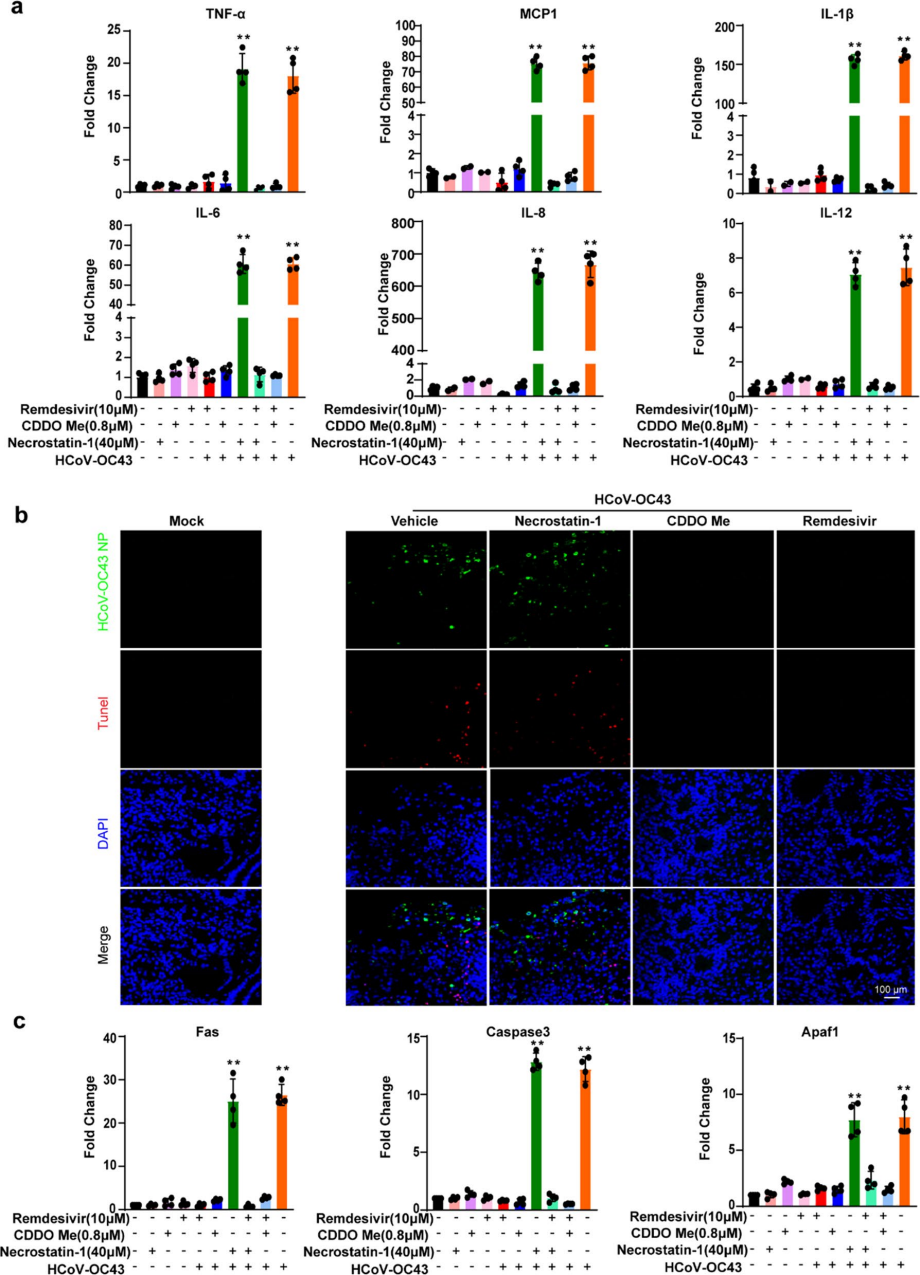
Remdesivir and CDDO-Me effectively inhibit HCoV-OC43-induced inflammation response and cellular apoptosis in HCOs. **a**. RT-qPCR was used to detect the mRNA expression levels of intracellular inflammatory factors (*TNF-α*, *MCP1*, *IL-1β*, *IL-6*, *IL-8*, and *IL-12*) after HCoV-OC43 infection of HCOs for 4 days with the drug alone or in combination(*p* < 0.05). **b**. TUNEL method was used to detect the cell death in HCoV-OC43-infected HCOs treated with Remdesivir (1 µM), CDDO-Me (0.8 µM) and Necrostatin-1(NCE1, 40 µM) after 4 days. **c**. RT-qPCR was used to detect the mRNA expression levels of apoptosis-related genes *caspase3*, *Fas*, and *Apaf1* after HCoV-OC43 infection of HCOs for 4 days with the drug alone or in combination (*p* < 0.05) (n = 4)

We determined whether cellular inflammation and damage were mitigated by drug treatment. The results showed that treatment with remdesivir and CDDO-Me, either alone or in combination, reduced the relative expression levels of proinflammatory cytokines TNF-α, MCP-1, IL-1β, IL-6, IL-8, and IL-12. In contrast, NEC-1 treatment alone failed to reverse the inflammatory response triggered by HCoV-OC43 infection (Fig. 6a). Furthermore, a reduction in cell death induced by HCoV-OC43 infection was observed after treatment with remdesivir and CDDO-Me (Fig. 6b). The expression of the apoptosis-related genes Caspase 3, Fas, and Apaf1 was significantly decreased following treatment with remdesivir and CDDO-Me (Fig. 6c). These results indicate that remdesivir and CDDO-Me effectively prevent the inflammatory response and cell death induced by HCoV-OC43 infection, whereas NEC-1 alone does not exert anti-inflammatory effects.

### HCoV-OC43 induces blood–brain barrier (BBB) injury

It is evident that HCoV-OC43 can infect HCOs, triggering inflammatory responses and cell death. Remdesivir and CDDO-Me effectively suppress HCoV-OC43 replication in HCOs and attenuate the associated inflammatory responses and cell death. To further investigate whether HCoV-OC43 infection disrupts the BBB, we established a co-culture model of the BBB and HCOs (Supplemental Fig. 5a). The TEER of the BBB was monitored for 14 consecutive days. The co-culture model maintained a stable TEER value above 150 Ω/cm^2^, indicating high barrier integrity (Supplemental Fig. 5b). Electron microscopy revealed the structural organization of the BBB, with HBECs located on the upper side of the transwell chamber and astrocytes together with pericytes on the lower side of the transwell chamber (Supplemental Fig. 5c). Immunofluorescence analysis further confirmed that the BBB structure comprised endothelial cells, astrocytes, and pericytes, which is consistent with the reported BBB model phenotype (Supplemental Fig. 5d). Collectively, these findings demonstrate the successful construction of a BBB–HCOs co-culture model.

**Fig. 7:**
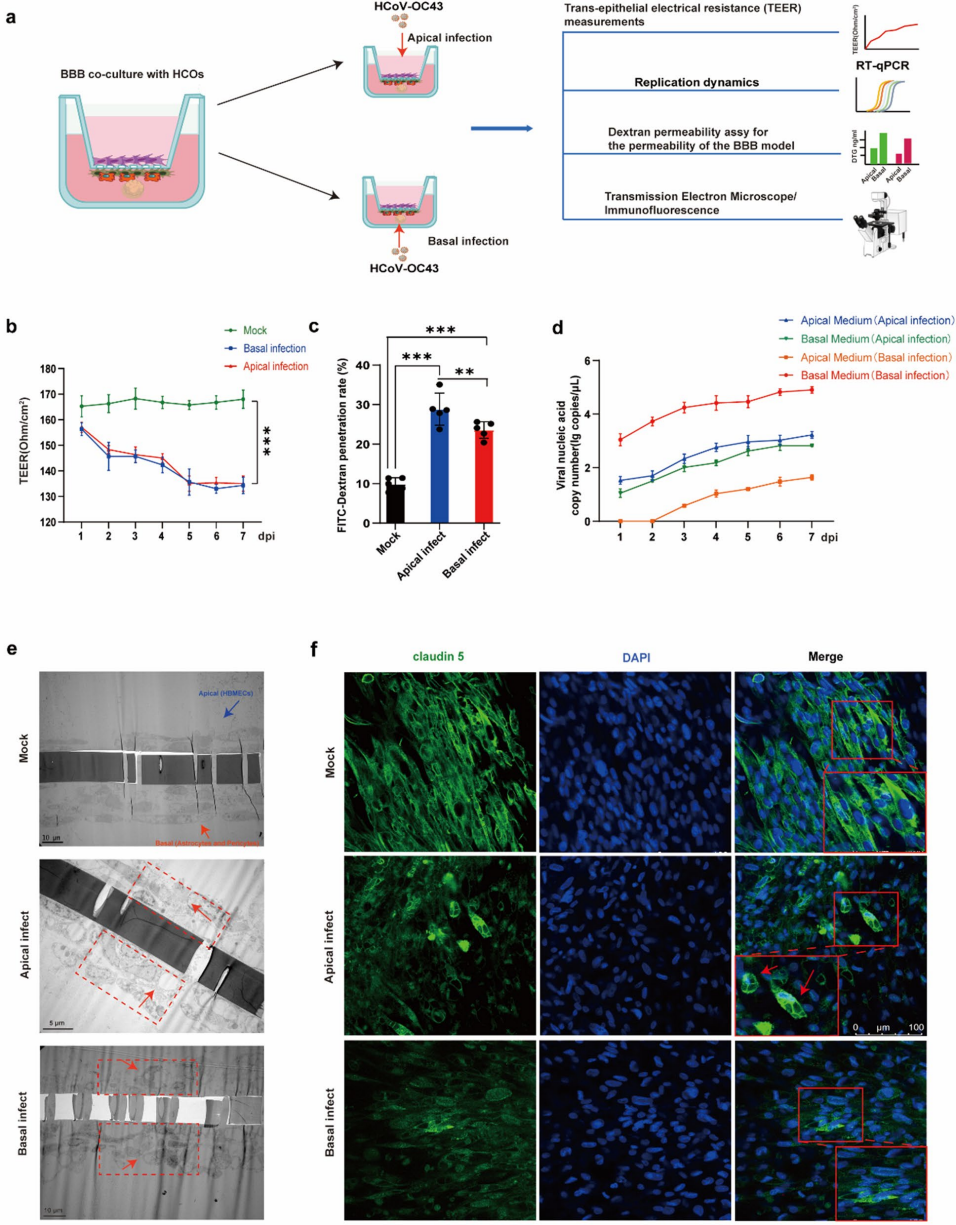
HCoV-OC43 infection disrupts the blood–brain barrier (BBB). **a**. Overview diagram of the co-culture model of BBB and HCOs infected with HCoV-OC43. **b**. Detect the TEER value of the co-culture model of BBB and HCOs infected with HCoV-OC43. **c**. Detect the BBB penetration rate of FITC-Dextran after HCoV-OC43 infection of the co-culture model of BBB and HCOs. **d**. Viral nucleic acid copy numbers detected in cell culture supernatant following HCoV-OC43 infection of the co-culture model of BBB and HCOs. **e**. The BBB structure of the HCoV-OC43 infection of the co-culture model of BBB and HCOs was observed by electron microscopy. **f**. Immunofluorescence was used to detect the expression of BBB tight junction proteins(claudin-5) in the HCoV-OC43 infection of the co-culture model of BBB and HCOs

Next, we infected the co-culture model with HCoV-OC43 from apical or basal infections respectively, and employed multiple assays to evaluate infection characteristics and associated damage (Fig. 7a). The results showed that HCoV-OC43 infection, whether on the apical or basal side, led to a decrease in the TEER value of the co-culture model (Fig. 7b), indicating that HCoV-OC43 infection compromised the integrity of the BBB. Infection with HCoV-OC43 resulted in increased BBB permeability to FITC-Dextran, with infection from HBECs side (apical) leading to higher permeability compared to infection from HCOs side (basal) (Fig. 7c). In the apical infection group, the viral nucleic acid copy number in the apical medium exhibited a time-dependent increase, which was similarly observed in the basal medium. These findings suggest that HCoV-OC43 can infect HBECs and subsequently traverse the blood–brain barrier (BBB) to basal medium. In the basal infection group, the viral nucleic acid copy number in the basal medium also increased with prolonged infection, and viral nucleic acid became detectable in the apical medium over time. This suggests that HCoV-OC43 infection compromises the integrity of the blood–brain barrier (BBB), as illustrated in Fig. 7d. We conducted separate infections of HBECs and astrocyte monolayers with HCoV-OC43. The results reaffirmed that HCoV-OC43 is capable of efficiently replicating in both cell types, as shown in Supplemental Fig. 6a. Furthermore, electron microscopy demonstrated that HCoV-OC43 infection in the co-culture model resulted in structural damage to BBB cells, characterized by vacuolar changes within these cells **(**Fig. 7e**)**. Immunofluorescence analysis revealed that HCoV-OC43 infection leads to the loss of tight junctions in HBECs, disrupted the organization of HBECs, and induced Syncytium -like structures within the HBECs **(**Fig. 7f**)**. Cytopathic effects (CPE) were observed after HCoV-OC43 infected endothelial cells and astrocytes respectively (Supplemental Fig. 6b). These findings demonstrate that HCoV-OC43 replicates within HBECs, induces BBB injury, and compromises barrier integrity.

## Discussion

HCoV-OC43 infection primarily affects the respiratory system; however, it has increasingly been associated with fatal encephalitis in immunodeficient children and elderly immunocompromised patients [8–12], as well as neurological disorders in adults [13–17]. Nevertheless, there is currently no human physiological model that demonstrates the direct replication of HCoV-OC43 in the human central nervous system. In this study, we developed and validated a hiPSC-derived human cerebral organoid (HCOs). Cerebral organoids are self-assembled in vitro from human induced pluripotent stem cells, effectively recapitulate key aspects of in vivo brain development. Mature cerebral organoids reproduce developmental hallmarks of the human brain, including ventricular zone (VZ) structures that house apical radial glia, subventricular zone (SVZ) areas containing intermediate progenitors and outer radial glia, and an emerging cortical plate (CP) populated with neurons [42]. For the first time, we have demonstrated that HCoV-OC43 can target and infect human neurons and astrocytes, and replicate in HCOs effectively.

The pathological findings of fatal encephalitis caused by HCoV-OC43 infection are consistent with the lesions observed in vitro during HCOs infection. The pathological manifestations in the central nervous system of a 10-month-old boy with multiple co-morbidities included meningoencephalitis affecting the cerebrum, cerebellum, brainstem, and spinomedullary junction. Microscopic examination revealed significant neuronal loss in the cortex, diencephalon, and basal ganglia, aligning with the observed neuronal tropism of HCoV-OC43 in HCO models [8]. An autopsy of an 11-month-old boy with severe combined immunodeficiency revealed considerable vacuolation in the cerebral cortex, accompanied by frequent nuclear dissolution of neurons [9]. The HCoV-OC43 nucleocapsid protein was detected in both neuronal and nerve fiber layers. Utilizing the HCO model, our study demonstrated that HCoV-OC43 is particularly susceptible to cortical neurons, inducing cell death and activating inflammatory responses, findings that are largely consistent with clinical cases. Notably, the infection of cerebral organoids by SARS-CoV-2 and the observed pathological brain damage in fatalities due to COVID-19 exhibit significant similarities across several dimensions. Autopsy findings reveal considerable neuronal damage and cell death in the brains of COVID-19 patients, manifested as hypoxic changes in neurons, erythrocyte degeneration, and apoptosis [43]. The infection of brain organoids by SARS-CoV-2 leads to neuronal cell death, characterized by highly condensed or fragmented nuclei and positive TUNEL staining [31]. Additionally, autopsy studies identify abnormal phosphorylation of Tau protein in the brains of COVID-19 patients, particularly in virus-positive neurons. The infection of cerebral organoids by SARS-CoV-2 also results in abnormal Tau protein phosphorylation in neurons, paralleling the abnormal phosphorylation observed in autopsy findings [39]. These results suggest that HCOs can serve as a human physiological model demonstrating the direct replication of human coronaviruses in the human central nervous system.

Our results indicated that HCoV-OC43 infection HCOs activates inflammatory responses and induces cell death through the TNF and NF-κB signaling pathways. We found that Remdesivir and CDDO-Me effectively inhibit viral replication in HCOs while alleviating inflammation and cell death. Furthermore, we confirmed that HCoV-OC43 directly infects HCOs and disrupts the blood–brain barrier (BBB). Once the virus breaches this barrier, it can subsequently infect HCOs. Conversely, when HCoV-OC43 directly infects HCOs, it can also penetrate the BBB, thereby impairing barrier function.

Recent perspectives suggested that HCoV-OC43 would be utilized to better understand the neurological impact of COVID-19 [44, 45]. Since both HCoV-OC43 and SARS-CoV-2 belong to the β-coronavirus genus, HCoV-OC43 shares similar genetic makeup with SARS-CoV-2. The spike proteins of HCoV-OC43 and SARS-CoV-2 share 41% sequence homology, both viruses bind to 9-O-acetylated sialic acids [46]. Our data showed that HCoV-OC43 can infect neurons and astrocytes within HCOs, replicating effectively and aligning with the results from the 2D cell and animal models [19–21]. Research showed that SARS-CoV-2 can infect neurons and astrocytes in brain organoids [31, 39, 40], suggesting that HCoV-OC43 and SARS-CoV-2 have similar affinity for targeting cells. Meanwhile, we found HCoV-OC43 NP protein was distributed along the axons of neurons, this is consistent with the results of HCoV-OC43 infection in the central nervous system of mice, suggesting that axonal transport enables the interneuronal transmission of HCoV-OC43 [47]. No syncytium formation was observed following HCoV-OC43 infection of HCOs, which was dramatically different with SARS-CoV-2 infection [44]. This discrepancy may be attributed to differences in the spike proteins of HCoV-OC43 and SARS-CoV-2 [48]. Interestingly, we found that HCoV-OC43 infected HCOs, it induced the death of adjacent cells of infected cells, which was similar to the result of SARS-CoV-2 infected HCOs [31], indicating that HCoV-OC43 and SARS-CoV-2 infected nerve cells to form a microenvironment or secrete some mediators, resulting in the death of adjacent cells.

In addition, our data indicate HCoV-OC43-induced defects at the molecular level, which may be related to brain tissue damage and patients with encephalitis. This finding is also similar to SARS-CoV-2 infection. Firstly, activation of TNF, NF-κB, NOD-like receptor, and chemokine signaling pathways, as well as pathways related to viral protein interactions with cytokines and receptors signaling stood out in an exploratory analysis of RNA-seq data, indicating inflammation as a factor contributing to brain tissue damage. Previous studies have shown that SARS-CoV-2 activates antiviral responses and the expression of inflammatory chemokines after infecting brain organoids, with upregulation of the NOD2 gene [31]. These results align with our findings regarding HCoV-OC43 infection in HCOs. Upregulation of NOD-like receptors promotes the activation of NF-κB and mitogen-activated protein kinase (MAPK), thereby stimulating the production of proinflammatory cytokines and chemokines [49]. NOD sensing by HCoV-OC43 may be key in coordinating the observed proinflammatory and innate immune responses. Secondly, downregulation of GABAergic synaptic and calcium signaling pathways may be associated with neuronal damage. Overall, our results support the view of HCoV-OC43 as a model for SARS-CoV-2 research [44].

At present, the treatment of viral encephalitis is mainly supportive treatment. In this study, we selected antiviral drugs and inhibitors that target the activated inflammatory pathways, the data showed that remdesivir and CDDO-Me effectively inhibited HCoV-OC43 replication while alleviating inflammation and cell death, confirming the potential of antiviral and anti-inflammatory drugs, either alone or in combination, to inhibit viral replication in HCOs and mitigate virus-induced inflammation and cell death. Our study is the first to demonstrate that CDDO-Me can inhibit HCoV-OC43 replication in HCOs while reducing inflammation and cell death. CDDO-Me, an antioxidant inflammation modulator and one of the most potent activators of nuclear factor erythroid 2-related factor 2 (Nrf2), activates multiple anti-inflammatory pathways through Nrf2, restoring redox balance and protein homeostasis, suppressing inflammation, and promoting tissue repair [50]. Previous studies have shown that CDDO-Me can inhibit the replication of rabies virus [51], influenza A virus [52], SARS-CoV-2 [53, 54], hepatitis B virus [55], and hepatitis C virus [56]. These results suggest that CDDO-Me has the potential for broad-spectrum antiviral activity. However, in clinical practice, it is often observed that neurological symptoms in HCoV-infected patients may be linked to long-term inflammatory responses and immune dysregulation persisting after the initial infection [56]. Therefore, a deeper understanding of the pathogenic mechanisms underlying virus-induced inflammation will be crucial for developing more effective drug strategies and improving current clinical treatments.

The CNS is protected by the BBB, which also constitutes a potential route and target for viral invasion. Viruses may also enter the CNS via perineuronal pathways [57]. Christian Bleau et al. reported that mouse hepatitis virus strains cross the BBB in a virulence-dependent manner, impairing barrier function and increasing permeability [58]. For SARS-CoV-2, infection of human brain microvascular cells increases BBB permeability by downregulating tight junction proteins [59]. Studies using microphysiological systems based on alveolar and BBB tissue chips revealed that direct exposure of BBB chips to SARS-CoV-2 caused only minor changes, whereas exposure to conditioned medium from infected alveolar chips led to more severe BBB damage, including endothelial dysfunction, pericellular detachment, and neuroinflammation [60]. Moreover, the SARS-CoV-2 spike protein alone has been confirmed to compromise BBB integrity [61, 62]. To investigate whether HCoV-OC43 similarly disrupts the BBB, we employed a BBB–HCOs co-culture model to simulate two infection modes. Our results showed that HCoV-OC43 replicates within HBECs, causing tight junction loss and functional impairment of the BBB. Likewise, HCoV-OC43 infection of HCOs disrupted both the structure and function of the BBB. Taken together, these findings demonstrate that HCoV-OC43 can penetrate the BBB via multiple infection routes, resulting in barrier dysfunction, and further support the use of HCoV-OC43 as a relevant research model for studying SARS-CoV-2 neuropathogenesis.

In conclusion, our data demonstrate that HCOs are an effective tool for studying neurotropic and neuroinvasive viruses, exploring the pathogenic mechanisms underlying viral encephalitis, and developing preventive and therapeutic strategies. Meanwhile, HCoV-OC43 infection model can be used as an alternative model of SARS-CoV-2 to further study the pathogenic mechanism of highly pathogenic coronavirus on human nervous system. However, this study has some limitations. Although the HCOs in this study contained various types of neural cells and complex neural networks, this model lacked immune cells [42]. Additionally, compared with the adult brain, our HCOs exhibited a lower degree of maturity, resembling fetal brain tissue [63, 64]. Furthermore, this study did not fully elucidate the specific molecular mechanisms by which HCoV-OC43 infection leads to encephalitis and breaks through the BBB. Therefore, investigating the precise pathogenic mechanisms is warranted. This will provide a foundation for understanding the pathogenic mechanisms of HCoV-OC43 in the central nervous system and offer new strategies for developing treatments for viral encephalitis.

## Conclusions

Our study reveals that HCoV-OC43 replicates efficiently within HCOs, primarily targeting neurons and astrocytes. HCoV-OC43 infection of HCOs might trigger an inflammatory response via the TNF and NF-κB signalling pathways, resulting in cellular death. Remdesivir and bardoxolone methyl (CDDO-Me) effectively an antiviral agent, inhibited HCoV-OC43 replication and reduced virus-induced inflammation and cell death. HCoV-OC43 replicates in HBECs, disrupts tight junctions, and impairs BBB function. Moreover, infection of HCOs by HCoV-OC43 can also penetrate the BBB, leading to barrier damage.

## Supplementary Information

Additional file 1.

## Data Availability

The datasets used and analyzed during the current study are available from the corresponding authors on reasonable request.

## Abbreviations

HCOs: Human cerebral organoids
ACE2: Angiotensin-converting enzyme 2
MS: multiple sclerosis
CDDO-Me: Bardoxolone methyl
CNS: Central nervous system
HCoVs: Human coronaviruses
hiPSC: Human induced pluripotent stem cell
ZIKV: Zika virus
HSV-1: Herpes simplex virus type I
SARS-CoV-2: Severe acute respiratory syndrome coronavirus 2
JEVs: Japanese encephalitis virus
NEC-1: Necrostatin-1
Nrf2: Nuclear factor erythroid 2-related factor 2
BBB: Blood–brain barrier
