# Supplementary material for: Human coronavirus OC43 infection in human cerebral organoids: novel insights on pathogenesis and potential therapeutic interventions
Source: J Biomed Sci. 2025 Nov 5;32:96. doi: 10.1186/s12929-025-01193-z (PMC12590701; doi:10.1186/s12929-025-01193-z)
Supplement: Supplementary file 1 — Additional file 1. [file 12929_2025_1193_MOESM1_ESM.docx]

**Supplementary materials**

**Fig.S1. Differentiation and identification of HCOs.** a. Schematic overview of HCOs generation using iPS cells. Bottom: representative images of organoids at different developmental stages. b. Representative images of 45-day-old organoids derived from iPS cells, showing markers for astroglia (GFAP), neural stem cells (Ki-67), neuronal progenitor cells (SOX2), neurons (TUJ1/MAP2), and deep layer cortical neurons (CTIP2). c. mRNA expression levels of various cell markers detected using RT-qPCR (n = 4).


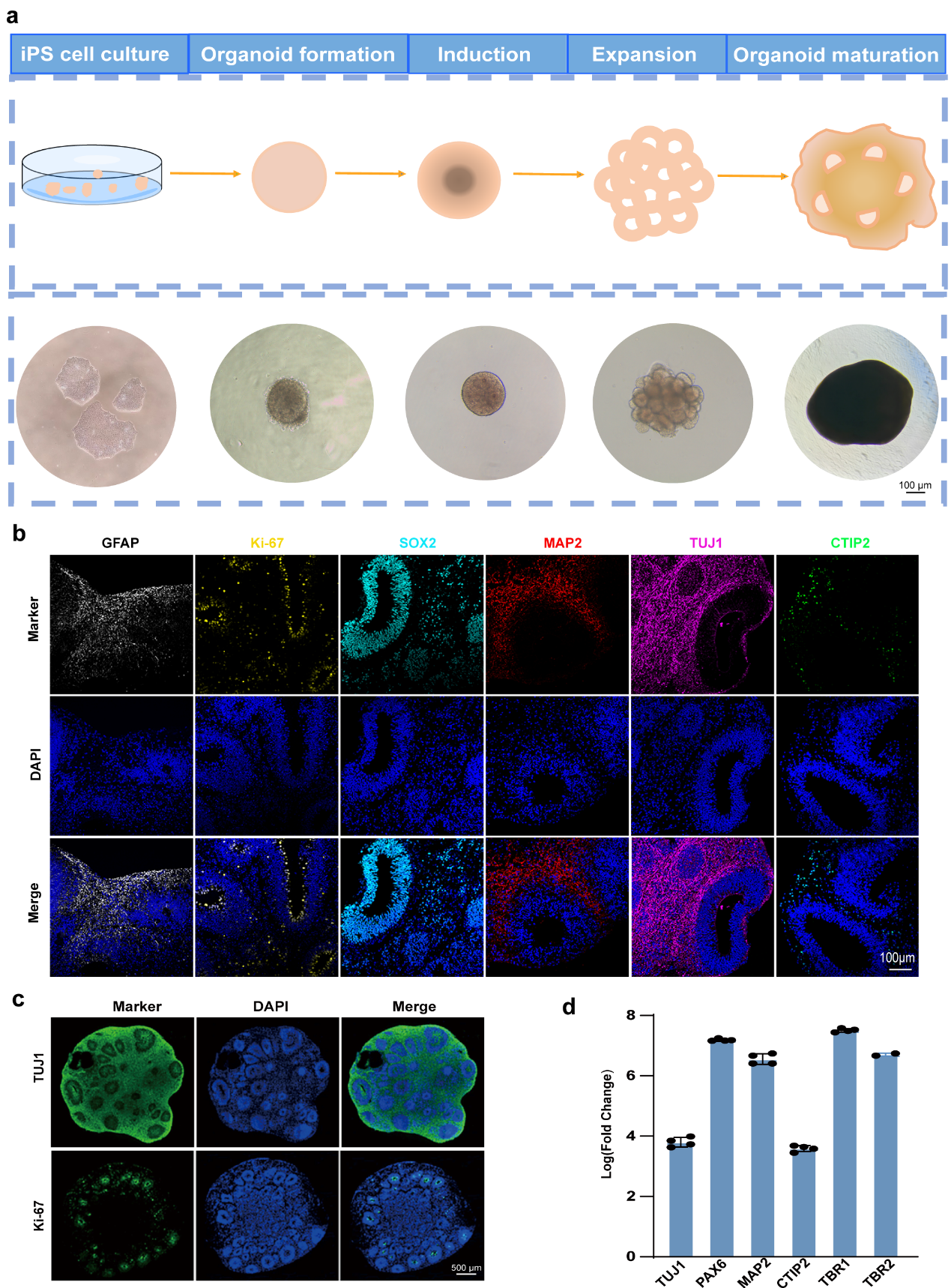


**Fig.S2 HCoV-OC43 does not infect neural progenitor cells in HCOs.**


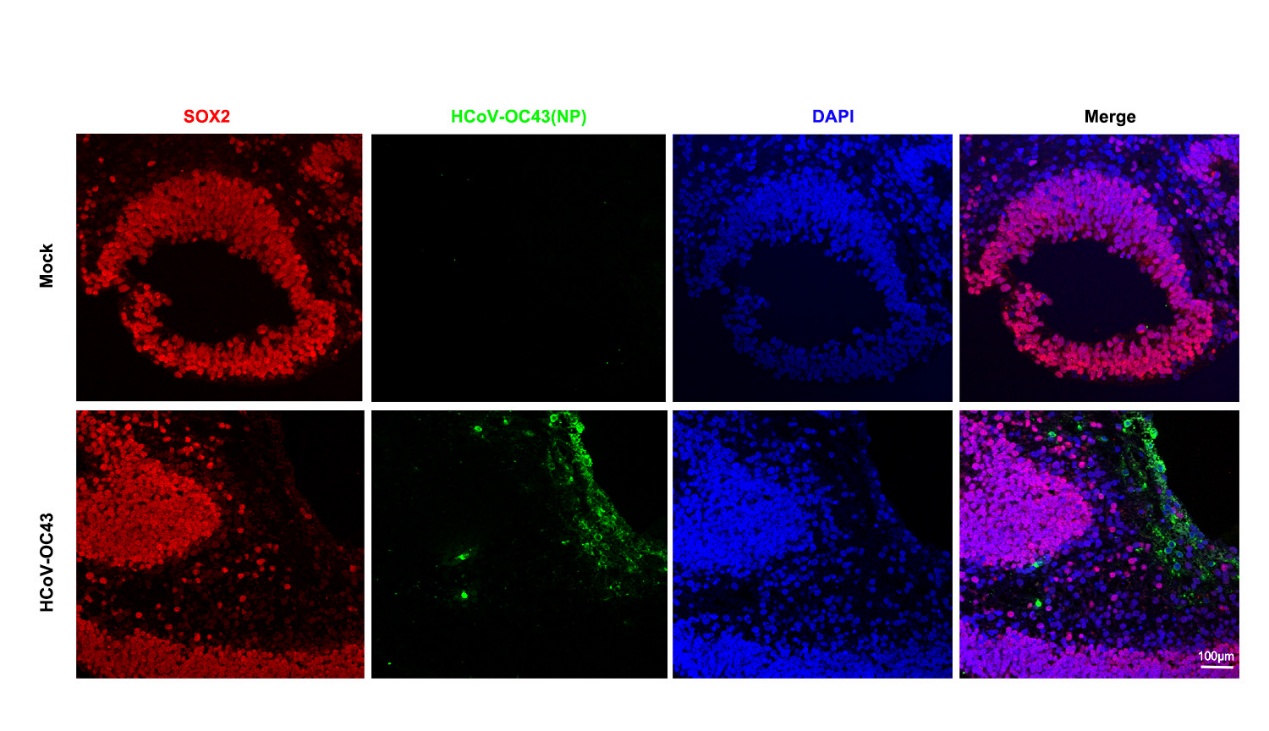


**Fig.S3 RNA-Seq analysis results show that genes related to inflammation and cell death are significantly upregulated after HCoV-OC43 infection in HCOs**.


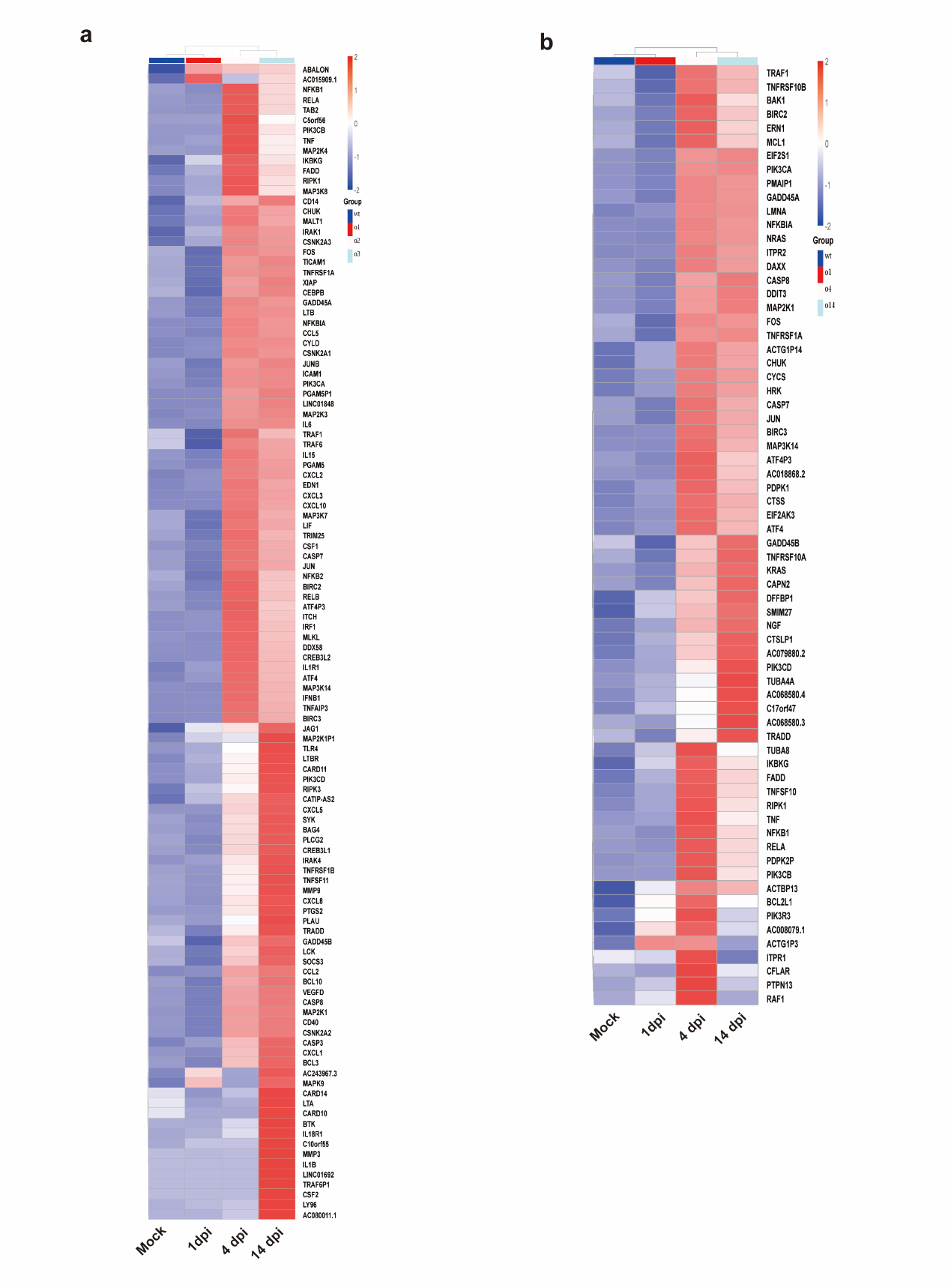


**Fig.S4**. The antiviral effect of remdesivir was dose-dependent.

**
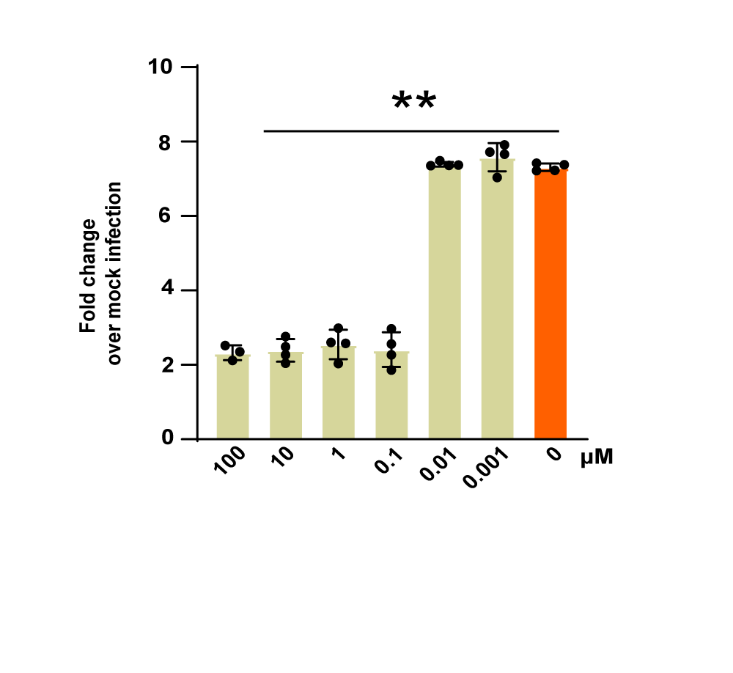
**

**Fig.S5 Establishment and identification of a co-culture model of blood-brain barrier (BBB) and HCOs.** a. The BBB establishment process, obtain the co-culture model of BBB and HCOs. b. The TEER values of the co-culture model of BBB and HCOs were continuously detected for 14 days. c. The structure of the BBB model was observed by electron microscopy. d. Immunofluorescence was used to identify various cell types in the BBB model: HBECs (claudin 5), Pericytes (CD146), Astrocytes (GFAP).


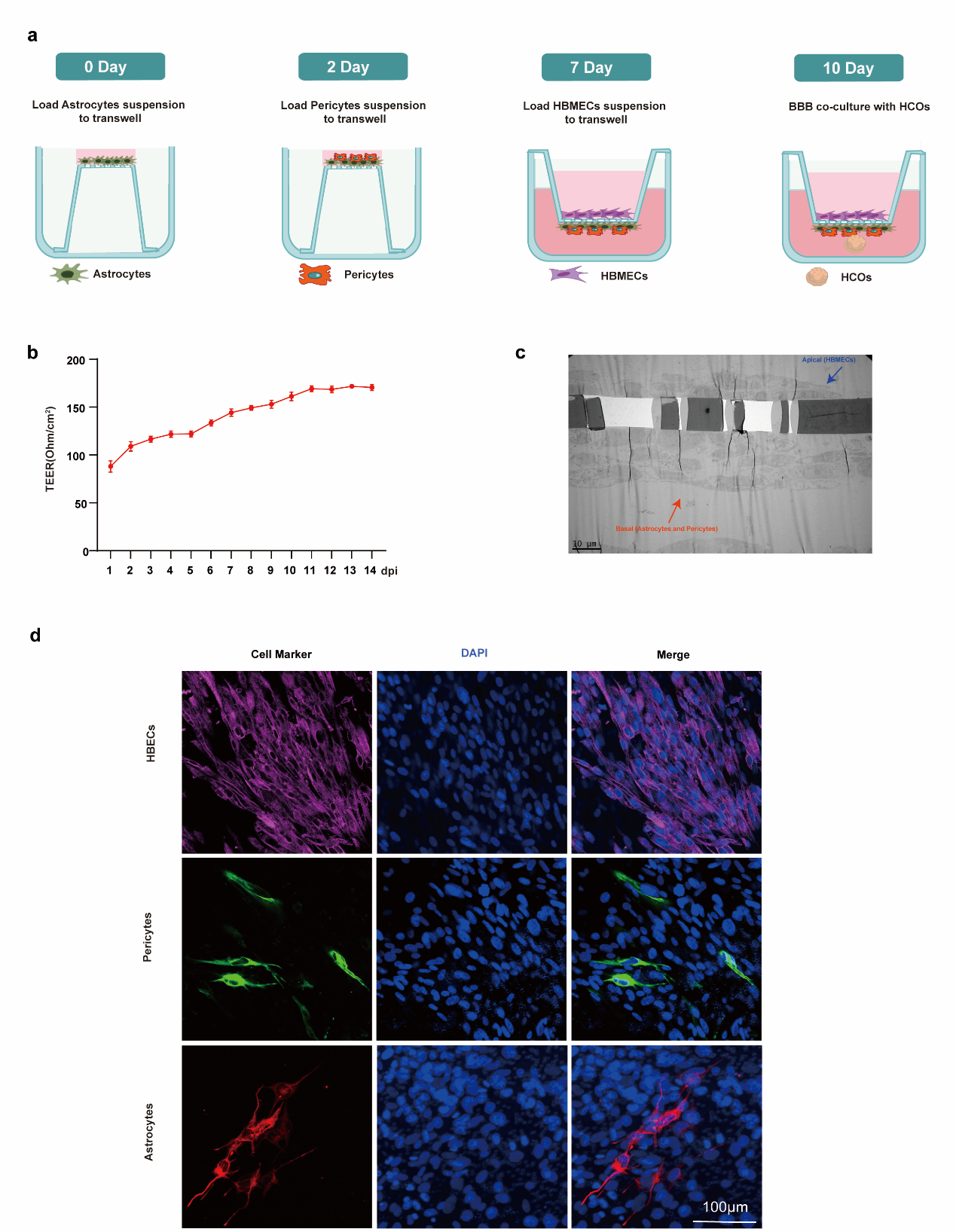


**Fig.S6 HCoV-OC43 replicates in HBECs and astrocytes.** a. Viral nucleic acid copy numbers detected in cell culture supernatant following HCoV-OC43 infection of the HBMECs and Astrocytes. b. Observe the CPE under a light microscope.


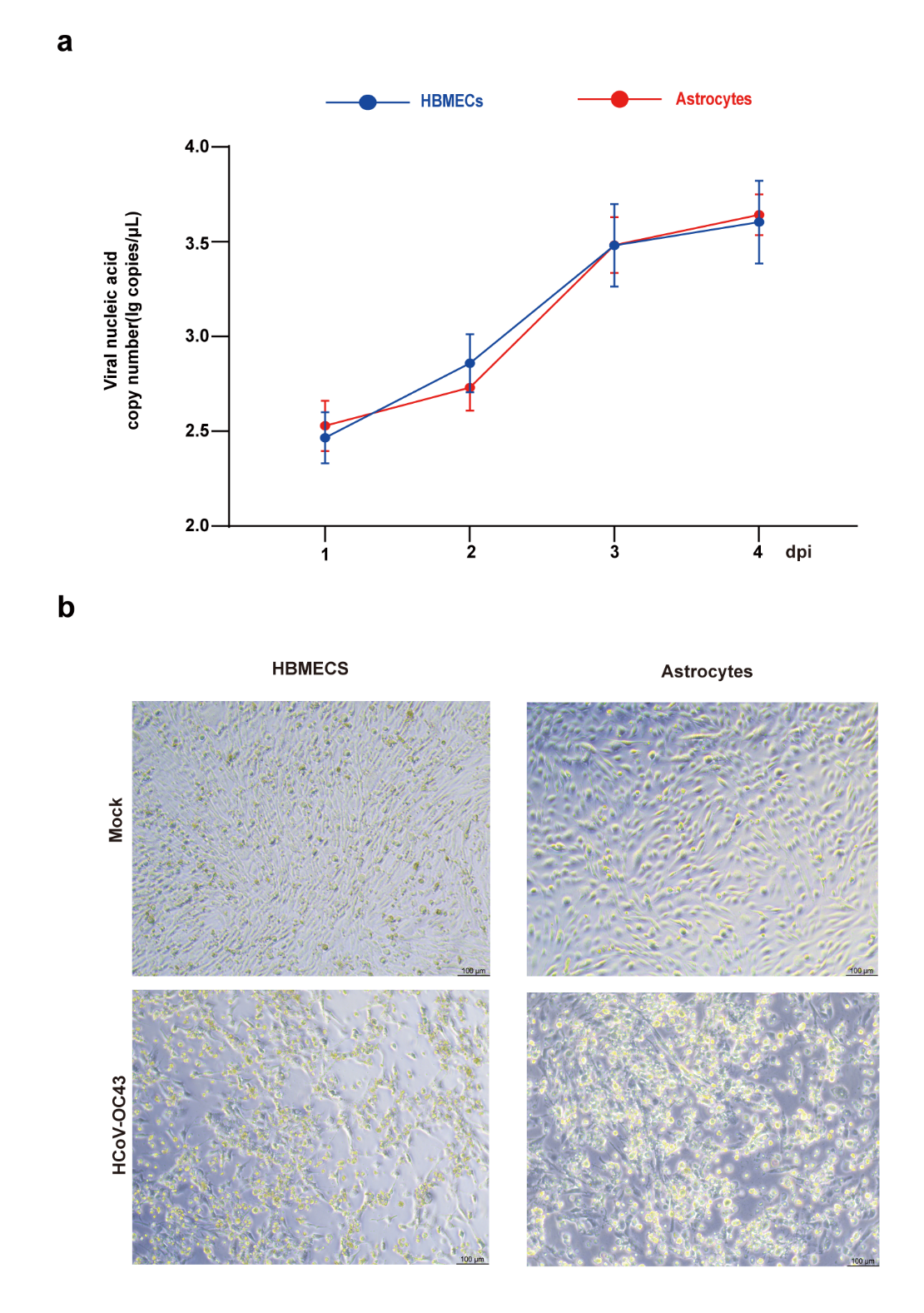


**Table S1 Primer sequence.**

| Primer | Forward | Reverse |
| --- | --- | --- |
| Fas | CACACTCACCAGCAACACCAAG | TGTACTTCCTTTCTCTTCACCCAAAC |
| Apaf-1 | ATGGCATTCCTGTTGTCTCTTCTTC | CATCCTGGTTCACCTTTCAATTTGG |
| Caspase3 | GAACTGGACTGTGGCATTGAGAC | AATAATAACCAGGTGCTGTGGAGTATG |
| TNF-α | AGCCCTGGTATGAGCCCATCTATC | TCCCAAAGTAGACCTGCCCAGAC |
| IL-6 | CCACACCAAGTTGAGGGAATGAGG | GAGTAGTGAGGAACAAGCCAGAGC |
| IL-1β | GGACAGGATATGGAGCAACAAGTGG | TCATCTTTCAACACGCAGGACAGG |
| IL-12 | CCTTGTGGCTACCCTGGTCCTC | AGTGGTGAAGGCATGGGAACATTC |
| IL-8 | CTCTCTTGGCAGCCTTCCTGATTTC | GGGGTGGAAAGGTTTGGAGTATGTC |
| MCP1 | ACCAGCAGCAAGTGTCCCAAAG | TTTGCTTGTCCAGGTGGTCCATG |
